# Management of work stress among arts and science school administrators in Nigeria using rational emotive occupational health coaching: A randomized controlled trial evaluation

**DOI:** 10.1097/MD.0000000000038135

**Published:** 2024-05-17

**Authors:** Ngozi Hope Chinweuba, Baptista Chinyere Chigbu, Assumpta C. Aham, Ifeoma E. Onyi, Nneka Chinyere Ezeugo, Blessing C. Anakpua, Regina Ijeamasi Enebechi, Ijeoma Awa Kalu, Nneka Justina Eze, Christian Sunday Ugwuanyi

**Affiliations:** a University of Nigeria, Nsukka, Nigeria; b Department of Science Education, University of Nigeria, Nsukka, Nigeria; c Nnamdi Azikiwe University, Awka, Nigeria; d Department of Science Education, Alex Ekwueme Federal University, Ndufu-Alike, Ebonyi State, Nigeria; e Department of Science Education, Nnamdi Azikiwe University, Awka, Nigeria; f Department of Educational Foundations, Alex Ekwueme Federal University, Ndufu-Alike, Ebonyi State, Nigeria; g Department of Linguistics, Foreign and Nigerian Languages, National Open University of Nigeria, Abuja, Nigeria; h University of the Free State, South Africa.

**Keywords:** administrators of secondary schools, arts and science, rational emotive occupational health coaching, work stress

## Abstract

**Background:**

The working circumstances of the administrators are appalling due to the nature of education in Nigeria. These administrators put in a lot of overtime to fulfill the expectations of their positions, which stresses them out. But there is no information in the literature about how administrators of science schools deal with their demanding environments. Therefore, the aim of this study was to evaluate how administrators of secondary scientific schools in the Southeast could manage work-related stress by using rational and emotive occupational health coaching.

**Methods:**

A randomized controlled trial (RCT) experimental design was used for the investigation, with 106 people divided into 2 groups–one for the intervention and one for the control. A selection of these participants came from southeast Nigerian special scientific schools. The Occupational Stress Index (OSI) and the Perceived Stress Scale (PSS) served as the foundation for our data collection procedure. A posttest was given following the 12-week intervention, and then there was a 2-month follow-up assessment. Repeated analysis of variance (ANOVA) was utilized to ascertain the effects both within and across groups.

**Results:**

It was revealed that rational emotive occupational health coaching had significant effect on the management of work stress among southeast secondary arts and science school administrators, *F* (2, 208) = 1452.484, *P* = <.050, ŋ^2^ = .933, and *F* (1, 104) = 18076.988, *P* = <.050, ŋ^2^ = .994).

**Conclusion:**

The management of work stress among southeast secondary arts and science school administrators was significantly improved through rational emotive occupational health coaching.

## 1. Introduction

In recent years, academics have experienced work stress due to the academic environment.^[1,2]^ Academics in higher education are under a lot of stress because of their administrative responsibilities.^[3]^ Teachers use technology to carry out their tasks, which can be stressful.^[4]^ Numerous studies have found that most workers’ stress is caused by working conditions that are harmful to their health^[5]^ and Onasoga et al^[6]^ Nigerian workplaces are incredibly stressful.^[7]^ Nwokeoma et al^[8]^ state that stress is one element that jeopardizes the productivity of Nigerian workers.

Workplace stress has been shown to have a negative effect on job performance, absenteeism, and increased workplace dangers.^[9]^ Workers are affected differentially by work stress due to variances in organizational commitments.^[10,11]^ High levels of stress at work are reported by academics from Australia and New Zealand,^[12]^ Canada,^[13]^ the United Kingdom,^[2,14]^ South Africa,^[4,15]^ and Nigeria.^[16–19]^ Besides, 39.25% of Nigerian workers experience stress at work.^[20]^ Studies have shown that this outcome is consistent with earlier research.^[7,21]^ Furthermore,^[22]^ discovered that roughly 10% of Nigerian employees experience extreme stress at work. Rational-emotional occupational health coaching (REOHC) can be used to help Nigerian workers manage their stress at work.

A REOHC intervention aims to help workers cope with work-related stress.^[23]^ REOHC interventions include physical group sessions or e-mail and WhatsApp chat applications.^[24]^ A negative perception of workplace climate may inhibit employees’ ability to demonstrate positive emotions, which ultimately limits their performance on the job, according to REOHC.^[24]^ Positive therapeutic relationships influence positive outcomes in REBT psychotherapy.^[25]^ In the management of troublesome personality traits, REBT therapy can be effective.^[26]^ Several adverse personality traits may be alleviated by REBT disputing, according to Blau et al^[27]^ REBT therapy provides a safe, protected environment for clients according to Garfield, Dempsey, Lamon, Sunderland et al and Blau, Fuller, Vaccaro.^[25–27]^

REBT therapy uses the ABCDE model as a tool for countering irrational beliefs and emotional reactions associated with one’s relationship to the environment.^[23]^ In order to explain why people develop irrational beliefs after having preferred goals blocked, Ellis proposed the ABCDE model. The triggering event for stress, worry, or a change in emotion is termed “A” by Ellis.^[28]^ A wide range of topics could be included, from the trivial to the significant. “B” stands for a person’s beliefs, or their cognitive responses to events. “C” represents repetitive, self-fulfilling consequences from an emotional standpoint. It is necessary to challenge irrational or limiting beliefs in order to bring about mental change. The letter “D” stands for dispute. “E” represents doing away with self-defeating beliefs. Cognitive restructuring involves creating new mental patterns and habits.

Numerous empirical studies have shown that counseling therapies can help manage work-related stress. Children who have experienced adverse childhood stress can significantly reduce irrational thoughts resulting from these experiences through rational emotive behavior therapy.^[29]^ According to Ogbuanya et al,^[24]^ occupational stress was significantly reduced in the REBC group compared to the waitlist control group. In a recent study, Eseadi et al^[30]^ reported that depression significantly decreased among participants in a rational emotive cognitive behavior coaching intervention. A significant drop in illogical career beliefs was observed when individuals received rational emotive behavior therapy compared to those who did not receive it, according to Ogbuanya et al^[31]^ Undergraduates’ burnout symptoms were reduced by rational-emotional behavior coaching, according to Ezenwaji et al^[32]^ Workers who work regularly in high-stress environments can often manage their subjective well-being through reasonable and empathetic occupational health coaching.^[23]^ It is evident from the above findings that REBT decreases irrational thoughts in a significant way. Police officers in Nigeria working in chronically stressful environments reported improved subjective well-being after receiving REOHC.^[8]^ In Nigerian schools, arts and science school administrators and police officers both work under stressful conditions.^[33]^ In conclusion, administrators of science schools will continue to be under stressful working conditions if they do not receive REBT interventions like REOHC. Southeast Nigerian secondary school administrators are responsible for managing schools properly and attending conferences. In spite of the fact that arts and science school administrators are well aware of the stress that comes with these various responsibilities, they are able to fulfill their duties effectively. As a result of the work stress of arts and science school administrators, students are not properly prepared for healthy living in society, or are not learning what they need to know to be successful in society.

There are no studies that have evaluated the effectiveness of REOHC in managing work stress among Nigerian arts and science school administrators. Consequently, there was a gap in literature about the effectiveness of REOHC in managing administrative staff work-related stress in Nigeria. In this study, we examined how REOHC affects the management of work stress among secondary arts and science school administrators in southeast Nigeria. Among arts and science school administrators, REOHC was hypothesized to significantly reduce work stress.

## 2. Methods

### 2.1. Ethical approval

In this study, permission was obtained from the Faculty of Education’s Ethics Committee on Research to conduct the research. Before beginning the intervention, participants were required to fill out informed consent forms.

### 2.2. Design of the study

The experimental design adopted was randomized controlled trial (RCT). Randomization-based trials are those in which participants are assigned at random to receive one of several distinct treatment therapies. A standard of comparison or control is one of these interventions. Following the interventions, the RCT measured and compared the outcomes.

### 2.3. Participants

**Figure 1.**
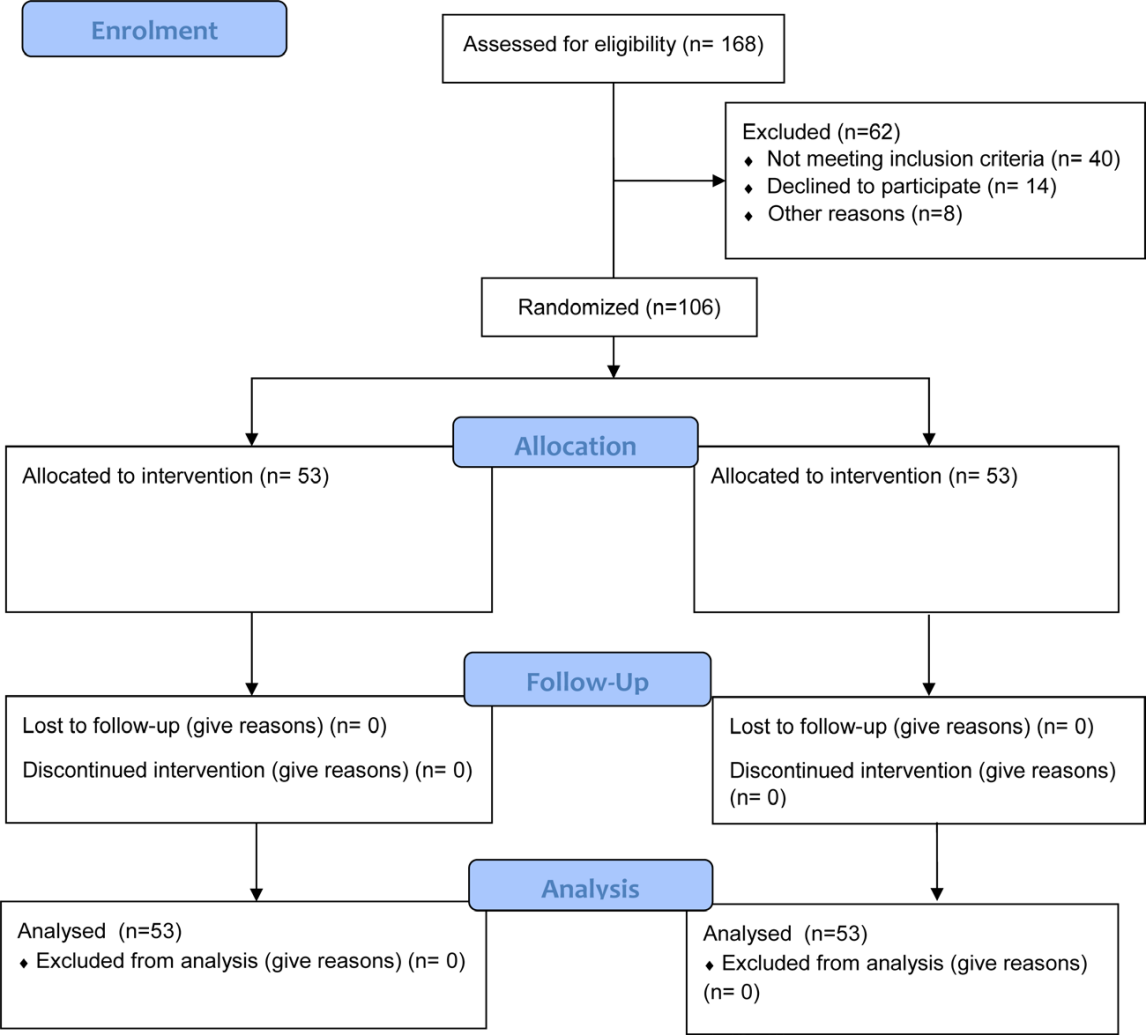
Flow diagram of the participants.

In southeast Nigeria, 106 secondary arts and science school administrators were randomly selected. Southeast has 5 states namely: Abai, Anambra, Ebonyi, Enugu and Imo. Participants were invited to express their interest to be considered for the intervention program. Following the conclusion of the advertisement period, 168 arts and science school administrators offered their time to participate in the intervention program. To determine eligibility for participation, the following criteria were used: Employees of special science secondary schools within SE Nigeria must be confirmed, Stress symptoms must be apparent after the baseline assessment of the occupational stress index (OSI). Five (5) years of experience as a principal or vice principal is required. Thus, 106 participants were selected for the study based on the eligibility criteria after checking for eligibility. The sample size for this study was 106, based on G-Power version 3.1, with a medium effect size (f2) of .15, a level of significance of .05, and a power of .91. Having a power of .91 was considered adequate.^[34]^ In addition to group size, independent variables and dependent variables were taken into account in determining the sample size. Fifty-three participants were randomly allocated to the intervention group, and 53 participants to the control group. Figure 1 displays the flow diagram for participants.

### 2.4. Measures

#### 2.4.1. Occupational stress index

In the study, an OSI developed by^[35]^ was used. Using this 46-item scale, employees are able to assess their level of stress within their work and personal lives. A response of 5 indicates absolute truth, 4 indicates almost truth, 3 indicates partially truth, 2 indicates almost false, and 1 indicates absolutely false. Worker’s occupational stress was estimated by adding the scores on all statements. Scores below 115 indicate low occupational stress, scores between 116 and 161 indicate moderate occupational stress, and scores above 161 indicate highly stressed workers. In terms of internal consistency, OSI items have a reliability coefficient of 0.79.

#### 2.4.2. Perceived stress scale

For this study, the Perceived Stress Scale (PSS) developed by^[36]^ was used. An individual’s perceived stress is measured with PSS, an instrument composed of ten items that are self-reported and unidimensional. Most psychological instruments used to measure stress perception are based on the Perceived Stress Scale. An individual’s perception of how stressful situations are in their life is measured by this scale. The respondents were asked to describe their lives as unpredictable, uncontrollable, and overloaded. A 5-point scale was used for the PSS items, with None (0), Almost Never (1), Sometimes (2), Fairly Often (3), and Very Often (4) being the lowest. A score of 0 is the lowest possible and a score of 40 is the highest. The participants were questioned about their attitudes, ideas, and behaviors concerning their homes and workplaces. Some instances are as follows: How many times in the last month have you been unhappy about something that came as a surprise? PSS items have an internal consistency reliability of 0.87.

### 2.5. Procedure

A declaration of interest in participation was advertised before the start of the intervention program. A total of 168 arts and science school administrators expressed interest in participating in the program through that channel. Using the set criteria for eligibility, the researchers administered the OSI to those who accepted to take part in the program. 106 participants met the eligibility criteria based on the selection process. A second measure was obtained through the administration of PSS to selected participants. Groups of intervention and control participants were then randomly assigned. Blinding of the randomization process was ensured by the researchers. A proper briefing was given to both groups regarding the study’s objectives and how the program would be implemented. WhatsApp online group conferencing was used for both REOHC and normal counseling interventions. The arrangement was made to provide participants with data bundles to motivate them and ensure their participation.

During the 12-week meeting period, we scheduled 2 6 to 8 pm meetings a week, one on Tuesday and one on Thursday. In the intervention group, participants were exposed to REOHC using the ABCDE model, whereas, in the nonintervention group, they received normal conventional counseling. To the intervention group, the conventional counseling served as a placebo. After the intervention period, 19/05/2023 to 19/08/2023, a 1-month follow-up period was conducted on 16/09/2023. Only PSS was administered to participants at the end of the program as posttest. Thus, PSS was administered thrice (pretest, posttest and follow-up) in this research. To determine the level of retention of the effect of the REOHC on the participants, a follow-up measure was obtained using PSS 2 months after the intervention.

### 2.6. Intervention

This study used a manual adapted from^[23,37]^ as outlined by Ellis and Grieger.^[35]^ As part of the manual, arts and science school administrators were given therapeutic strategies for becoming their coaches. The researchers in the occupational health coaching intervention found cognitive, affective, and emotive techniques to be helpful in coping with participants’ work stress. The purpose of REBT treatment was to change target irrational beliefs using cognitive (e.g., disputation), behavioral, and emotive techniques. One meeting was held twice a week for 12 weeks during the 12-week clinical trial.^[37,38]^ In phase 1 of the 12-week treatment period, weeks 1 to 4 were divided into 4 weeks. In phase 2, weeks 5 to 8 were divided into 4 weeks. In phase 3, weeks 9 to 12 were divided into 4 weeks.

#### 2.6.1. Phase 1: Weeks 1 to 4

Participants were introduced to the intervention, familiarized, and clarified the aspects in the first meeting. The list of stress-related issues that the participants have is part of this phase, which also involves developing a therapeutic alliance through empathy, REBT training, setting expectations for the intervention, and conceptualizing a general intervention. As the intervention progresses, the participants and the intervention team check the status of their therapeutic relationship throughout the process of building 1.

#### 2.6.2. Phase 2: Weeks 5 to 8

Before the start of phase 2, participants discussed assignments and homework assigned during previous sessions. During this phase, participants were told to bolster their rational views while reducing their irrational ones. We urged participants to recognize links across issues, especially those marked by shared irrational beliefs, by downplaying some of the illogical beliefs they held to be possible stresses. Therefore, in order to encourage a stress-free work atmosphere, rational self-beliefs were emphasized. The participants were given homework assignments at the conclusion of each section.

#### 2.6.3. Phase 3: Weeks 9 to 12

The final phase of the program, which lasted 4 weeks, prepared the participants to become their future therapists or coaches. Participants were notified and discussed their assignments and homework from phase 2. This phase also discussed the first session’s relapse prevention structure and dependency problems. In similar studies,^[23,24]^ the ABCDE model of REBT was used.

### 2.7. Method of data analysis

The statistical analysis was conducted using statistical package for social sciences version 22. Prior to carrying out the actual analysis of the data, errors were checked, standard processes were established, accuracy was validated, and duplicates were scoured. The effects of within-groups and between-groups were determined using mixed design repeated measures analysis of variances (ANOVA). In repeated measures ANOVA, the assumption of sphericity states that there is no difference in variance between any pair of within-subject conditions. Mauchly test of sphericity was performed to determine whether the assumption of sphericity was violated, and the result was not significant (Mauchly *W* = .854, *P* = .412).

## 3. Results

Table 1 demonstrates that the number of male and female science school administrative staff who took part in the study did not differ significantly (χ^2^[1] = 1.98, *P* = .072). Age and religion of the participants, however, varied significantly: χ^2^(2) = 18.65, *P* < .050; χ^2^(2) = 15.98, *P* < .050; χ^2^(4) = 21.75, *P* < .050, and χ^2^(1) = 19.06, *P* < .050.

**Table 1:** Demographic characteristics of the participants.

Demographics		Intervention group n (%)	Nonintervention group n (%)	χ^2^	*P*
Gender	Male	36 (67.92)	34 (64.15)		
	Female	17 (32.08)	19 (35.84)	1.06	.093
Age	30–40 yrs	8 (15.09)	11 (20.75)	8.35	<.05
	41–50 yrs	24 (45.28)	27 (50.94)		
	51 yrs and above	21 (39.62)	15 (28.30)		
Religion	Christian	48 (90.57)	39 (73.58)	34.89	<.05
	Moslem	5 (9.43)	14 (26.42)		

Report of the baseline assessment using OSI.

Table 2 shows that at the baseline assessment, the mean OSI of the intervention group participants (*M* = 165.07, *SD* = 6.98) did not differ significantly (*t* [104] = 1.021, *P* = .879) from the mean OSI of the nonintervention group participants (*M* = 164.99, *SD* = 7.01).

**Table 2:** Mean analysis of the OSI scores of the participants.

Baseline measures					
Treatment	n	Mean	*SD*	t	*P*
Intervention	53	165.07	6.98	1.021	.879
nonintervention	53	164.99	7.01		

Table 3 demonstrated that, as determined by PSS (*M* = 35.81, *SD* = .1.66), the mean perceived stress rating of the intervention group’s participants was nearly identical (*t* [104] = 15.87, *P* = .00) to that of the nonintervention group’s participants (*M* = 35.73, *SD* = 1.67), *t* (104) = .76, *P* = .98. On the other hand, the intervention group’s mean perceived stress rating (*M* = 13.00, *SD* = 2.29) at the posttest was lower (*t* [104] = 15.87, *P* = .00) than the nonintervention group’s (*M* = 34.43, *SD* = 3.51). Additionally, at the follow-up assessment, the intervention group’s participants’ mean perceived stress rating (*M* = 12.52, *SD* = 2.33) was lower (*t* [104] = 16.89, *P* = .00) than that of the nonintervention group (*M* = 34.24, *SD* = 3.56),

**Table 3:** Mean analysis of the work stress ratings of the participants.

Treatment	n	Pretest (1)				Posttest (2)				Follow-up (3)			
		Mean	SD	t	*P*	Mean	SD	t	*P*	Mean	SD	t	*P*
Intervention	53	35.81	1.66	.76	.98	13.00	2.29	15.87	.00	12.52	2.33	16.89	.00
nonintervention	53	35.73	1.67			34.43	3.51			34.24	3.56		

**Figure 2.**
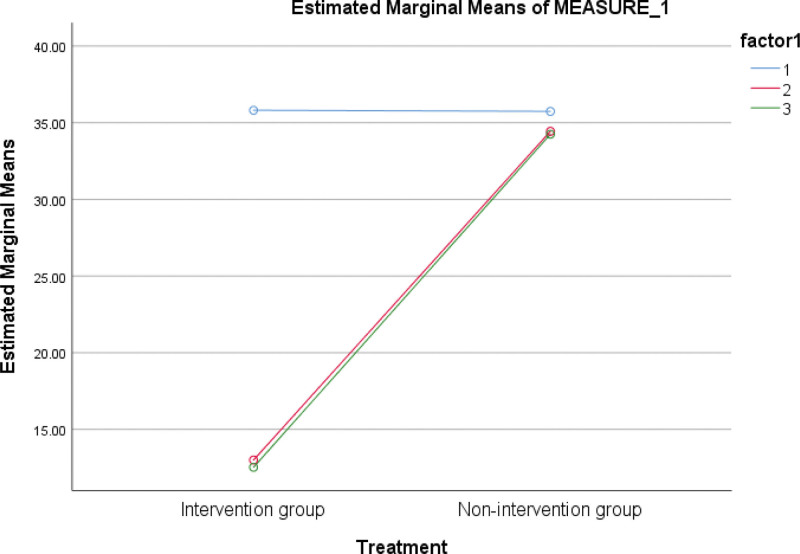
Interaction graph of time and treatment.

Table 4 shows that for the within-groups effect, the arts and science school administrators showed significant variations in work stress management throughout the 3-time measurements, *F* (2, 208) = 1452.484, *P* = <.050, ŋ^2^ = .933, and also for the between-groups effect, *F* (1, 104) = 18076.988, *P* = <.050, ŋ^2^ = .994). Additionally, a significant interaction effect was observed between the treatment and time, *F* (2, 208) = 1139.095, *P* < .050, and ŋ^2^ =.916 (see Fig. 2). Table 5 indicates that all measure pairs have mean differences that are significant at *P* < .050. This suggests that the considerable impact of time on the participants’ work stress was not influenced by mean differences between measures 2 and 3, 3 and 2.

**Table 4:** Within-subjects effect and between-subjects effects of the intervention using mixed design repeated measures.

Measure	Source		Type III sum of squares	df	Mean square	F	Sig.	Partial eta squared
Tests of within-subjects effect								
PSS	Time	Sphericity assumed	10561.252	2	5280.626	1452.484	.000	.933
	Time * Treatment	Sphericity assumed	8282.547	2	4141.274	1139.095	.000	.916
	Error (Time)	Sphericity assumed	756.201	208	3.636			
Tests of between-subjects effect								
PSS	Intercept		242692.531	1	242692.531	18076.988	.000	.994
	Treatment		16390.217	1	16390.217	1220.828	.000	.921
	Error		1396.252	104	13.425			

ŋ^2^ = Effect size.

**Table 5:** Pairwise comparisons.

(I) factor1	(J) factor1	Mean difference (I-J)	Std. error	Sig.^b^	95% Confidence interval for difference^b^	
					Lower bound	Upper bound
1	2	12.169*	.459	.000	11.038	13.300
	3	12.728*	.478	.000	11.550	13.906
2	1	−12.169*	.459	.000	−13.300	−11.038
	3	.559*	.192	.015	.087	1.032
3	1	−12.728*	.478	.000	−13.906	−11.550
	2	−.559*	.192	.015	−1.032	−.087

Based on estimated marginal means.

* . The mean difference is significant at the .05 level.

b .Adjustment for multiple comparisons: Bonferroni.

Subsequent analysis of this interaction revealed that there was no statistically significant difference between the baseline and that the nonintervention group’s ability to manage work-related stress did not change over time. On the other hand, the intervention group’s mean stress ratings gradually dropped, suggesting that REOHC had a major impact on the arts and science school administrators’ ability to handle work-related stress. Furthermore, the REOHC intervention was found to be responsible for 93.3 percent reduction in work stress experienced by arts and science school administrators, as evidenced by the effect sizes of .933.

## 4. Discussion of the findings

An examination of how REOHC can be used to manage work stress among southeast secondary arts and science school administrators was undertaken in this study. Researchers found that the average stress ratings of the intervention group decreased with time, indicating that REOHC significantly reduced work stress among secondary arts and science school administrators. The REOHC intervention program’s engaging nature can be explained by these findings. It has been empirically proven that the intervention program is capable of changing clients’ irrational thoughts to rational ones. Using the REBT, it can be argued that irrational beliefs can cause maladaptive emotions, thus reducing well-being.^[23]^ By using REBT, therapists can help workers broaden their understanding of work experiences. Thus, CBT improves positive affect and life satisfaction and decreases negative thinking/belief.^[39,40]^ It has been demonstrated that coaching increases stress resilience and performance by David et al^[41,42]^ Several studies substantiate the above points.^[8,23,24,29–32]^

Rational emotive behavior therapy’s cognitive restructuring intervention programs were successful in lessening irrational ideas connected to traumatic childhood experiences.^[29]^ Through the use of REOH treatment,. Additionally, Ogbuanya et al^[24]^ discovered that by lowering occupational stress and enhancing workability, rational emotive behavior coaching enhances the performance of Nigerian electronics workshop instructors. Following the rational emotive cognitive behavior coaching intervention, participants’ depression significantly decreased, according to Eseadi et al^[30]^ Onuigbo et al^[43]^ discovered that a rational emotive behavior therapy (REBT) method was useful for controlling stress at work.

In a similar study,^[31]^ reported that those receiving rational emotive behavior therapy significantly reduced their illogical career views as compared to those who were not receiving it. A group-focused rational emotive behavior coaching program was successful in lowering the symptoms of burnout in undergraduate students.^[44]^ In the same vein, cognitive behavior interventions have proved effective in similar studies.^[45–53]^ Similarly,^[8]^ discovered that rational emotive occupational health coaching significantly improved the work-related stress management of Nigerian Police officers as compared to their peers in the waiting-list control group. In order to manage their subjective well-being, rational emotive occupational health coaching proved to be useful in a study including police officers and workers who experience chronic stress. It affects how efficiently arts and science school administrators carry out their duties. This suggests that if REOHC doesn’t step in, administrators at different universities will be forced to operate in stressful environments without understanding how to handle the problem. According to Du Plessis,^[3]^ open distance learning institutions and universities rely significantly on technology to make sure that graduates are employable and can fulfill their students’ needs at any time of day. Thus, a major stressor in higher education is keeping up with information technology. In other words, sufficient use of REOHC in the various southeast Nigerian secondary schools from time to time will enable the arts and science school administrators to get more work done with less stress if they are counseled from time to time.

### 4.1. Limitations of the study

During the intervention period, poor browsing networks may have impeded the flow of intervention contents, so the generalizability of the results may be limited. Also, the researchers were unable to evaluate the effects of tribe, gender, religion and age on the impact of REOHC on work stress. Researchers suggested that future investigations duplicate the study in person and take into account any potential moderating factors on the effect of cognitive behavioral therapy on work-related stress in light of the findings.

### 4.2. Conclusion

The researchers concluded that rational emotive occupational health coaching is an effective intervention for managing work stress among arts and science school administrators. As the first research output that proves the efficacy of REOHC in work stress management among arts and science school administrators in the SE region of Nigeria, this contributes to the existing body of knowledge in the field of science and social science education. No empirical evidence existed on this topic before this research output. Using the REOHC intervention program, the researchers recommend that relevant school authorities organize seminars and workshops for arts and science school administrators to be counseled based on the study’s findings. It is important to organize this seminar or workshop periodically to enable arts and science school administrators to cope with the challenges they face.

## Acknowledgments

Throughout the study, all participants actively participated in the intervention program, which was appreciated by the researchers.

## Author contributions

**Conceptualization:** Ngozi Hope Chinweuba, Baptista Chinyere Chigbu, Assumpta C Aham, Ifeoma E. Onyi, Nneka Chinyere Ezeugo, Blessing C Anakpua, Regina Ijeamasi Enebechi, Ijeoma Awa Kalu, Nneka Justina Eze, Christian Sunday Ugwuanyi.

**Data curation:** Christian Sunday Ugwuanyi.

**Formal analysis:** Christian Sunday Ugwuanyi.

**Funding acquisition:** Ngozi Hope Chinweuba, Baptista Chinyere Chigbu, Assumpta C Aham, Ifeoma E. Onyi, Nneka Chinyere Ezeugo, Blessing C Anakpua, Regina Ijeamasi Enebechi, Ijeoma Awa Kalu, Nneka Justina Eze, Christian Sunday Ugwuanyi.

**Investigation:** Ngozi Hope Chinweuba, Baptista Chinyere Chigbu, Assumpta C Aham, Ifeoma E. Onyi, Nneka Chinyere Ezeugo, Blessing C Anakpua, Regina Ijeamasi Enebechi, Ijeoma Awa Kalu, Nneka Justina Eze, Christian Sunday Ugwuanyi.

**Methodology:** Ngozi Hope Chinweuba, Baptista Chinyere Chigbu, Christian Sunday Ugwuanyi.

**Project administration:** Ngozi Hope Chinweuba, Baptista Chinyere Chigbu, Assumpta C Aham, Ifeoma E. Onyi, Nneka Chinyere Ezeugo, Blessing C Anakpua, Regina Ijeamasi Enebechi, Ijeoma Awa Kalu, Nneka Justina Eze, Christian Sunday Ugwuanyi.

**Resources:** Ngozi Hope Chinweuba, Baptista Chinyere Chigbu, Assumpta C Aham, Ifeoma E. Onyi, Nneka Chinyere Ezeugo, Blessing C Anakpua, Regina Ijeamasi Enebechi, Ijeoma Awa Kalu, Nneka Justina Eze, Christian Sunday Ugwuanyi.

**Software:** Christian Sunday Ugwuanyi.

**Supervision:** Ngozi Hope Chinweuba, Baptista Chinyere Chigbu, Ifeoma E. Onyi, Nneka Justina Eze, Christian Sunday Ugwuanyi.

**Validation:** Baptista Chinyere Chigbu, Christian Sunday Ugwuanyi.

**Visualization:** Ngozi Hope Chinweuba, Baptista Chinyere Chigbu, Christian Sunday Ugwuanyi.

**Writing – original draft:** Ngozi Hope Chinweuba, Baptista Chinyere Chigbu, Christian Sunday Ugwuanyi.

**Writing – review & editing:** Ngozi Hope Chinweuba, Baptista Chinyere Chigbu, Christian Sunday Ugwuanyi.
